# Vaccination with a chikungunya virus-like particle vaccine exacerbates disease in aged mice

**DOI:** 10.1371/journal.pntd.0007316

**Published:** 2019-04-26

**Authors:** Maria T. Arévalo, Ying Huang, Cheryl A. Jones, Ted M. Ross

**Affiliations:** 1 Center for Vaccines and Immunology, University of Georgia, Athens, GA, United States of America; 2 Department of Infectious Diseases, University of Georgia, Athens, GA, United States of America; Centers for Disease Control and Prevention, UNITED STATES

## Abstract

**Introduction:**

Chikungunya virus (CHIKV) is a re-emerging pathogen responsible for causing outbreaks of febrile disease accompanied with debilitating joint pain. Symptoms typically persist for two weeks, but more severe and chronic chikungunya illnesses have been reported, especially in the elderly. Currently, there are no licensed vaccines or antivirals against CHIKV available. In this study, we combined a CHIK virus-like particle (VLP) vaccine with different adjuvants to enhance immunogenicity and protection in both, adult and aged mice.

**Methods:**

CHIK VLP-based vaccines were tested in 6-8-week-old (adult) and 18-24-month-old (aged) female C57BL/6J mice. Formulations contained CHIK VLP alone or adjuvants: QuilA, R848, or Imject Alum. Mice were vaccinated three times via intramuscular injections. CHIKV-specific antibody responses were characterized by IgG subclass using ELISA, and by microneutralization assays. In addition, CHIKV infections were characterized in vaccinated and non-vaccinated adult mice and compared to aged mice.

**Results:**

In adult mice, CHIKV infection of the right hind foot induced significant swelling, which peaked by day 7 post-infection at approximately 170% of initial size. Viral titers peaked at 2.53 × 10^10^ CCID_50_/ml on day 2 post-infection. Mice vaccinated with CHIK VLP-based vaccines developed robust anti-CHIKV-specific IgG antibody responses that were capable of neutralizing CHIKV *in vitro*. CHIK VLP alone or CHIK plus QuilA administered by IM injections protected 100% of mice against CHIKV. In contrast, the antibody responses elicited by the VLP-based vaccines were attenuated in aged mice, with negligible neutralizing antibody titers detected. Unvaccinated, aged mice were resistant to CHIKV infection, while vaccination with CHIKV VLPs exacerbated disease.

**Conclusions:**

Unadjuvanted CHIK VLP vaccination elicits immune responses that protect 100% of adult mice against CHIKV infection. However, an improved vaccine/adjuvant combination is still necessary to enhance the protective immunity against CHIKV in the aged.

## Author summary

Chikungunya virus is responsible for outbreaks of febrile illnesses accompanied with debilitating join pain in subtropical and tropical regions of the world. The disease caused by chikungunya virus typically resolves itself within weeks, but may be persistent and more severe in elderly individuals. Currently, there are no licensed vaccines, although a virus-like particle vaccine is currently being tested in Phase II clinical trials. In this study, we formulated chikungunya virus-like particles with adjuvants to skew and enhance the immune responses against chikungunya, and vaccinated adult and aged mice. Our aim was to identify a vaccine formulation that would protect adult and elderly populations. Results showed that the unadjuvanted vaccine was very effective in adult mice, eliciting strong virus-neutralizing antibody titers, and protecting mice against chikungunya infection and disease. In contrast, chikungunya disease was exacerbated in mice vaccinated with the virus-like particle vaccine alone or with QuilA adjuvant. This study highlights the need for an improved vaccine approach to safely and effectively vaccinate the elderly against chikungunya viral infections.

## Introduction

Chikungunya virus (CHIKV) is a re-emerging pathogen responsible for causing outbreaks of febrile disease accompanied with debilitating joint pain. CHIKV was first discovered in Tanzania in 1952, but outbreaks became more widespread, encompassing countries in Africa, Asia, Europe, and islands of the Pacific and Indian Oceans before emerging in the Americas in 2013 [1, 2]. More recently, in 2016–2017, there has been a resurgence of autochthonous CHIKV transmission in India [3], Pakistan [4], and Italy [5]. The virus is transmitted to humans through the bite of *Aedes aegypti* and *Aedes albopictus* mosquitoes. Chikungunya infection results in illness, in which fever and joint polyalrthralgia, are typically reported symptoms [6]. Acute symptoms persist for two weeks, but more chronic arthralgias may persist for months to years in a subset of subjects. More severe and/or chronic chikungunya illnesses were first widely reported in retrospective studies of the chikungunya epidemics of Reunion Island [7, 8] and India [9]. Following infection, patients experience renal, respiratory, hepatic, and cardiovascular system failures. In addition, diseases of the central nervous disease and encephalitis are major areas of concerns [7–9]. People over 60 years of age are at particular risk for severe chikungunya-associated illnesses, with case fatalities reported [8–10]. However, the incidence of CHIKV infection in this population is not remarkable in comparison to other age groups [11–13]. The specific mechanisms that lead to increased severity of CHIKV illness in the elderly are not known, but increased understanding could lead to better treatments and vaccines for this at-risk population.

Vaccinating elderly individuals presents a special challenge since they are more prone to severe illness and vaccine efficacy drops in this population [14]. The age-associated changes in the immune system are collectively termed immunosenescence and include fewer circulating antigen presenting cells and tissue-associated dendritic cells, decreased phagocytosis, decreased toll-like receptor signaling, reduced naïve B and T cells, and chronic basal level of inflammation [15]. Elements of the immune system that remain intact include tissue macrophages and CD8+ T cell-mediated responses [14, 15]. Different vaccine approaches to counter immunosenescence in the aging include the use of higher vaccine doses, booster vaccinations, adjuvants, and vector-based vaccines [15]. Many vaccine delivery platforms are in development for a chikungunya vaccine, including formalin-inactivated viral vaccines, live-attenuated viruses, chimeric alphaviruses, DNA-based vaccines, recombinant subunit vaccines, and virus-like particle [VLP]-based vaccines [16]. The most promising candidates, including a non-adjuvanted CHIK VLP vaccine, are being tested in Phase I and II clinical trials in adults between the ages of 18–60 years of age [17]. Thus, we will continue to have a gap in knowledge regarding 1) CHIKV vaccine efficacy in the elderly and 2) understanding the vaccine characteristics needed to elicit a protective immune response in this population.

In this study, CHIKV virus-like particles were adjuvanated and used to vaccinate adult and aged mice. Adjuvants were chosen for their abilities to not only enhance, but skew immune responses. The goal was to identify a CHIK VLP vaccine formulation that would protect both adult and aged mice populations.

## Materials and methods

### Expression of Chikungunya E1, E2, and VLPs

The complete sequence encoding structural proteins (C-E3-E2-6K-E1) of the Chikungunya virus S27 strain [accession #AF369024] was codon-optimized for expression in *Spodoptera frugiperda* and synthesized by Genewiz [South Plainfield, NJ, USA]. The Bac-to-Bac baculovirus expression system [Thermo Fisher Scientific, Waltham, MA, USA] was subsequently used to generate recombinant baculoviruses expressing CHIKV structural proteins. Briefly, the structural gene sequence was inserted into the pFastBac1 vector, under the control of the *Autographa californica* multiple nuclear polyhedrosis virus (AcMNPV) polyhedrin for high-level expression in insect cells. The CHIK C-E VLP/pFastBac1 construct was then transformed into DH10Bac *E*. *coli*, where C-E genes flanked between mini-Tn7 sites on the pFastBac1 plasmid and the LacZ gene flanked between mini-*att*Tn7 target sites on a AcMNPV bacmid are transposed to generate recombinant bacmid. The presence of C-E genes was verified by polymerase chain reaction (PCR) analysis using primers that hybridize to sites flanking the mini-*att*Tn7 site: pUC/M13 forward 5’-CCCAGTCACGACGTTGTAAAACG-3’ and pUC/M13 reverse 5’-AGCGGATAACAATTTCACACAGG-3’. Baculovirus was generated and passaged in Sf9 *S*. *frugiperda* insect cells, maintained in serum-free, SF900 II SFM medium [Thermo Fisher Scientific]. To generate the initial recombinant viruses, 8×10^5^ Sf9 cells per well were seeded onto a 6-well plate and allowed to adhere for 15 min. The cells were then transfected with 1–2 μg bacmids using Cellfectin transfection reagent [Thermo Fisher Scientific]. The cells were observed for cytopathic effect and supernatants were harvested and clarified after 72h post-infection. The P1 virus was then passaged in a 30ml, spinner-flask culture of Sf9 cells at a cell density of 2×10^6^ c/ml, and harvested 72h post-infection to generate P2 virus. For expression, Sf9 cells were cultured in spinner flasks to a density of 2×10^6^ c/ml in a total volume of 250 ml and infected with recombinant baculovirus at a MOI of 1. Cultures were harvested once cell viability was reduced to roughly 80% or 72-96h after infection, and the cells were pelleted at 500×g for 5 min at 4°C. Supernatants were collected and filtered through a 0.22μm pore membrane before sedimentation via ultracentrifugation. CHIK virus-like particles (VLP) were sedimented through a 20% glycerol cushion at 100,000×g for 4h. The sedimented VLP pellets were resuspended in sterile phosphate buffered saline (PBS). Similarly, E1 and E2 genes, designed as transmembrane-truncated versions, were synthesized and cloned into the pFastBac HT vector. The pFastBac HT vector adds an N-terminal 6×His tag and and tobacco etch virus (TEV) proteolytic site to each gene. Recombinant bacmids and baculoviruses were generated as described above and soluble E1 and E2 proteins were expressed in Sf9 spinner flask cultures.

### Soluble E1 and E2 protein purification

The cultures containing soluble E1 and E2 proteins were harvested and cells were sedimented at 500×g for 5 min at 4°C. Supernatants were collected and filtered through a 0.22μm pore membrane and the proteins were purified by affinity chromatography using Ni-NTA resin [Thermo Fisher Scientific]. Briefly, the clarified cultures were incubated with Ni-NTA resin with shaking for 2.5-3h at room temperature before they were added to the columns. The medium was allowed to flow through and the Ni-NTA resin was washed three times with PBS containing 10mM imidazole. His-tagged proteins were then eluted twice with PBS containing 250mM imidazole. Upon verification of eluted proteins by SDS-PAGE analysis, E1 and E2 proteins were dialyzed and concentrated using Amicon Ultra-15 centrifugal filters [Millipore, Burlington, MA] with a 10 KDa molecular weight cut-off and sterile 10% glycerol in PBS as the exchange buffer. Total protein concentrations for E proteins and VLPs were measured using the Micro BCA protein kit as per manufacturer’s protocol [Pierce, Rockford, IL, USA].

### SDS-PAGE analysis of E1 and E2 purification fractions

Samples from each step of the purification process were prepared by combining 30μl of samples with 6μl 6×Laemmli buffer with beta-mercaptoethanol [βME] and heating to 100°C for 5 min. Proteins were separated on a Bolt 10% Bis-Tris Plus gel [Thermo Fisher Scientific] at 200V for 30 min and protein bands were stained with PageBlue protein staining solution [Thermo Fisher Scientific] and destained with distilled water.

### Western Blot

Samples were prepared by mixing 10μg of total protein in Laemmli buffer with βME, unless otherwise noted. These samples were boiled at 100°C for 5 min and proteins were separated on Bolt 10% Bis-Tris Plus gel as before. Next, the proteins were transferred from the gels onto PVDF membranes using the Trans Blot Turbo apparatus [Bio-Rad, Hercules, CA, USA]. The membranes were blocked for 5–10 min in iBind solution [Novex]. Polyclonal mouse anti-E1 and anti-E2 sera were recovered from mice vaccinated with E1 or E2 proteins in the lab and used to probe for these proteins. Mouse monoclonal antibody against E2 [Clone CHK-48, BEI Resources, Manassas, VA, USA] was also used to probe for E2. Goat anti-mouse conjugated with horseradish peroxidase [Southern Biotech, Birmingham, AL] was used as the secondary antibody. The membrane, antibody, and iBind solutions were loaded into the iBind Western System [Life Technologies, Carlsbad, CA] from which point all steps in the membrane blotting process proceed automatically by sequential lateral flow. Blotting using the iBind system was complete after 2.5 h. Following washing of the membrane twice more with PBS with 0.05% Tween-20 [PBS-T], the membrane was exposed with Clarity Western ECL Substrate [Bio-Rad]. Images were captured using my ECL Imager [Thermo Fisher Scientific].

### Cell culture and viruses

Vero cells [ATCC, Manassas, VA, USA] were cultured in Dulbecco’s Modification of Eagle’s Medium [DMEM, Mediatech, Manassas, VA, USA] supplemented with 10% fetal bovine serum [FBS], 2mM L-glutamine, 100 U/ml penicillin, and 100μg/ml streptomycin [10%FBS-DMEM] and maintained at 37°C and 5% CO_2_. C6/36 mosquito cells [ATCC] were cultured at 28°C and 5% CO_2_ in Eagle’s mimimum essential medium [EMEM, Mediatech] supplemented with 10%FBS, 2mM L-glutamine, 100 U/ml penicillin, and 100μg/ml streptomycin [10%FBS-MEM]. CHIKV LR2006-OPY1 virus was obtained from the World Reference Center for Emerging Viruses and Arboviruses (WRCEVA). Upon receipt, this virus was passaged twice in C6/36 mosquito cells. Virus concentration was determined in Vero cells and reported as the 50% cell culture infectious dose (CCID_50_) per volume [ml].

### Vaccinations

Female C57BL/6J mice were obtained from the Jackson Laboratory [Bar Harbor, ME, USA] at 6–8 weeks of age for studies in adult mice. Female C57BL/6J mice were also obtained at 12 months and allowed to age to at least 18 months for studies in aging mice. All procedures in the document were approved by the UGA Institutional Animal Care and Use Committee, # A2015 06-004-Y3-A12. Mice were immunized on days 0, 21, and 42 and blood samples were taken on days 0, 14, and 35 via the submandibular method using 5mm lancets.[18] Vaccines were formulated to contain 30μg [~0.3–0.4μg E2 content] chikungunya VLPs adjuvanted with 20μg QuilA [InvivoGen, San Diego, CA, USA], 10μg R848/Resiquimod [InvivoGen], 1:1 by volume Imject Alum [Thermo Fisher Scientific], or in PBS alone (no adjuvant). Vaccines were delivered via intramuscular injection to the hindlimb quadriceps in a total volume of 50μl or subcutaneous injection to the scruff of the neck in a total volume of 100μl.

### Detection of CHIKV-specific antibodies by enzyme-linked immunosorbent assay

Nunc Maxisorp 96-well plates [Thermo Fisher Scientific] were coated overnight at 4°C with 10μg/ml E1, E2, or VLP in PBS. The plates were then washed three times with PBS with 0.05% Tween-20 (PBS-T) and blocked with 200μl 1% bovine serum albumin in PBS (blocking buffer) for 1 hr at room temperature. Serum samples from individual mice were diluted to 1:100 in blocking buffer and added at 100μl/well in duplicate wells. The sera were allowed to react for 2 hr at room temperature. The plates were washed three times with PBS-T and bound sera were reacted with goat anti-mouse IgG-Fc [1:50,000], IgG1 [1:10,000], IgG2c [1:10,000], or IgG3 [1:10,000] antibody conjugated with alkaline phosphatase [Bethyl Laboratories, Montgomery, TX, USA] for 1 hr at room temperature. The plates were washed three more times and allowed to develop for 20 min following the addition of 100 μl para-nitrophenylphosphate substrate [SeraCare, Milford, MA, USA]. The plates were read at a wavelength of 405nm using a BioTek PowerWave XS plate reader with Gen5 version 2.07 software [BioTek, Winooski, VT, USA].

### Microneutralization assays

Mouse sera from immunized or naïve mice were heat-inactivated at 56°C for 30 min. Two-fold serial dilutions of the sera were prepared in 10% FBS-DMEM and added to 96-well, cell-culture plates. CHIKV LR2006-OPY1 strain was then added at 200 CCID_50_ per well, and virus-antibody solutions were incubated together for 1 h at 37°C and 5% CO_2_. The final serum dilutions ranged from 1:20 to 1:2560. Each plate had two sets of assay controls: one column of wells contained virus only and a second column contained medium only. Vero cells were added at 10^4^ cells/well and plates were incubated for 5 days at 37°C and 5% CO_2_. The cells were fixed for 20 min with 10% formalin in PBS and stained with crystal violet for 5 min at room temperature. Neutralizing titers were measured and expressed as the reciprocal of the highest serum dilution that inhibited cytopathic effect.

### Viral challenge

Adult C57BL/6J mice [6-8-week old] were challenged with CHIKV Reunion Island isolate LR2006-OPYI, which is of East Central South Africa lineage [ECSA] as previously described.[19] Mice were observed for 14 days following challenge. Prior to infection, the mice were anesthetized with a 100μl cocktail of 10 mg/kg xylazine plus 100 mg/kg ketamine in via intraperitoneal injection, and initial weight and hind foot measurements were recorded. Foot size was defined as width × breadth (mm^2^) and measured using a digital micrometer with 0.001mm resolution. The virus was subcutaneously injected into the right hind footpad at 50μl/mouse, while the mice were still under anesthesia. Pilot viral dose challenge experiments were conducted in naïve adult and aging mice to determine optimal challenge conditions. Mice were observed twice daily and weight and foot measurements were recorded once a day. Blood samples were collected on between days 1–5, and at day 14 post-infection. Based on these initial studies, a 10^5^ CCID_50_ challenge dose of LR2006-OPY1 CHIKV was used to test vaccine efficacy, and blood collections were reduced to 2, 4, 6, and 14 days post-infection. Approximately 40–60 μl of blood was collected from mice on sampling days, except on day 14 when the mice were anesthetized and terminally bled.

### Measurement of viral loads in sera

A two-step assay was used to measure viral loads in serum samples of mice challenged with CHIKV. C6/36 cells were grown to 100% confluence in T75 culture flasks, detached by scraping, and divided equally into four 96-well plates per T75 flask. The next day, ten-fold serial dilutions (10^−1^–10^−8^) of the mouse sera were prepared in 10%FBS-EMEM and used to inoculate 96-well plates of confluent C6/36 at 100μl /well. The cells were allowed to incubate for 3 days at 28°C and 5% CO_2._ Vero cells were seeded at 2×10^4^ cells/well in a total volume of 100μl 10% FBS-DMEM per well and 25μl of C6/36 culture supernatants were transferred into triplicate wells containing Vero cells. Vero cells were incubated for 4 days at 37°C and 5% CO_2_. The cells were then fixed with 10% formalin for 20 minutes and stained with 1% crystal violet solution for 5 minutes at RT. Cells with 95% or more cytopathic effect were counted for each dilution and viral loads [CCID_50_/ml] were calculated using the Spearman-Karber equation.[20]

### Inflammatory cytokine assay

Mouse TNF-α, IL-6 and IL-1β ELISA MAX kits from BioLegend [San Diego, CA, USA] were used to detect inflammatory cytokines in sera of adult and aged mice, as per manufacturer’s protocol. Briefly, Nunc Maxisorp plates were coated coated overnight at 4°C wit 100 μl/well of anti-mouse TNF-α, IL-6, or IL-1β diluted to 1:200 in carbonate buffer, pH 9.5. After washing once with PBS-T, all subsequent steps were performed at room temperature, with shaking. Plates were blocked with blocking buffer (1% BSA in PBS). TNF-α and IL-6 standards were diluted and used at final concentrations 3.9–500 pg/ml, while IL-1β was used at 15.6–2000 pg/ml in blocking buffer. Pooled sera from adult or aged naïve mice were prepared by mixing 10 individual serum samples together. Pooled sera and standards were added to plates [100 μl/well] and incubated for 2 hr. Plates were then washed four times with PBS-T and incubated with biotinylated detection antibody at 1:200 dilution in blocking buffer for 1 hr. The plates washed 4 times with washing buffer and 1:1000 diluted avidin-HRP was added and incubated for 30 mins. After 5 washes, TMB substrate solution was added (100μl/well) and plates were incubated in the dark for 15 mins. The reaction was stopped with 100μl/well stop solution (2N H_2_SO_4_) and plates were read using the PowerWave XS microplate spectrophotometer at a wavelength of 450 nm.

### TNF-α inhibition of CHIKV infections *in vitro*

Recombinant mouse TNF-α [Life Technologies] was diluted in 10% FBS-DMEM and mixed with 200 CCID_50_ CHIKV LR2006-OPY1 virus per well, and virus-antibody solutions were incubated together for 1 h at 37°C and 5% CO_2_. The final TNF-α dilutions ranged from 5–80 ρg/ml. Each plate had two sets of assay controls: one column of wells contained virus only and a second column contained medium only. Vero cells were added at 10^4^ cells/well and plates were incubated for 5 days at 37°C and 5% CO_2_. The cells were fixed for 20 min with 10% formalin in PBS and stained with crystal violet for 5 min at room temperature. Wells with 95% or more cytopathic effect were counted for each TNF-α dilution and reported as the percentage of wells with CPE.

### Statistical analyses

GraphPad Prism 7 for Mac OS X software was used to perform statistical analyses. One-way, two-tailed ANOVA, followed by Tukey post-hoc tests were performed for data derived from one time-point. Two-way, two-tailed ANOVA followed by post-hoc tests were performed for data collected over multiple time-points. A p-value of less than 0.05 was considered significant.

### Ethics statement

All mouse-related experiments were conducted in compliance with the guidelines of the University of Georgia Institutional Animal Care and Use Committee [A2015 06-004-Y3-A12], and in accordance with the National Research Council’s Guide for the Care and Use of Laboratory Animals, The Animal Welfare Act, and the CDC/NIH’s Biosafety in Microbiological and Biomedical Laboratories guide. Management of animal experiments, care, and was conducted by the University of Georgia’s Animal Resources Department that is accredited by the AAALAC.

## Results

### Production and purification of recombinant CHIK VLP and E proteins

**Fig 1:**
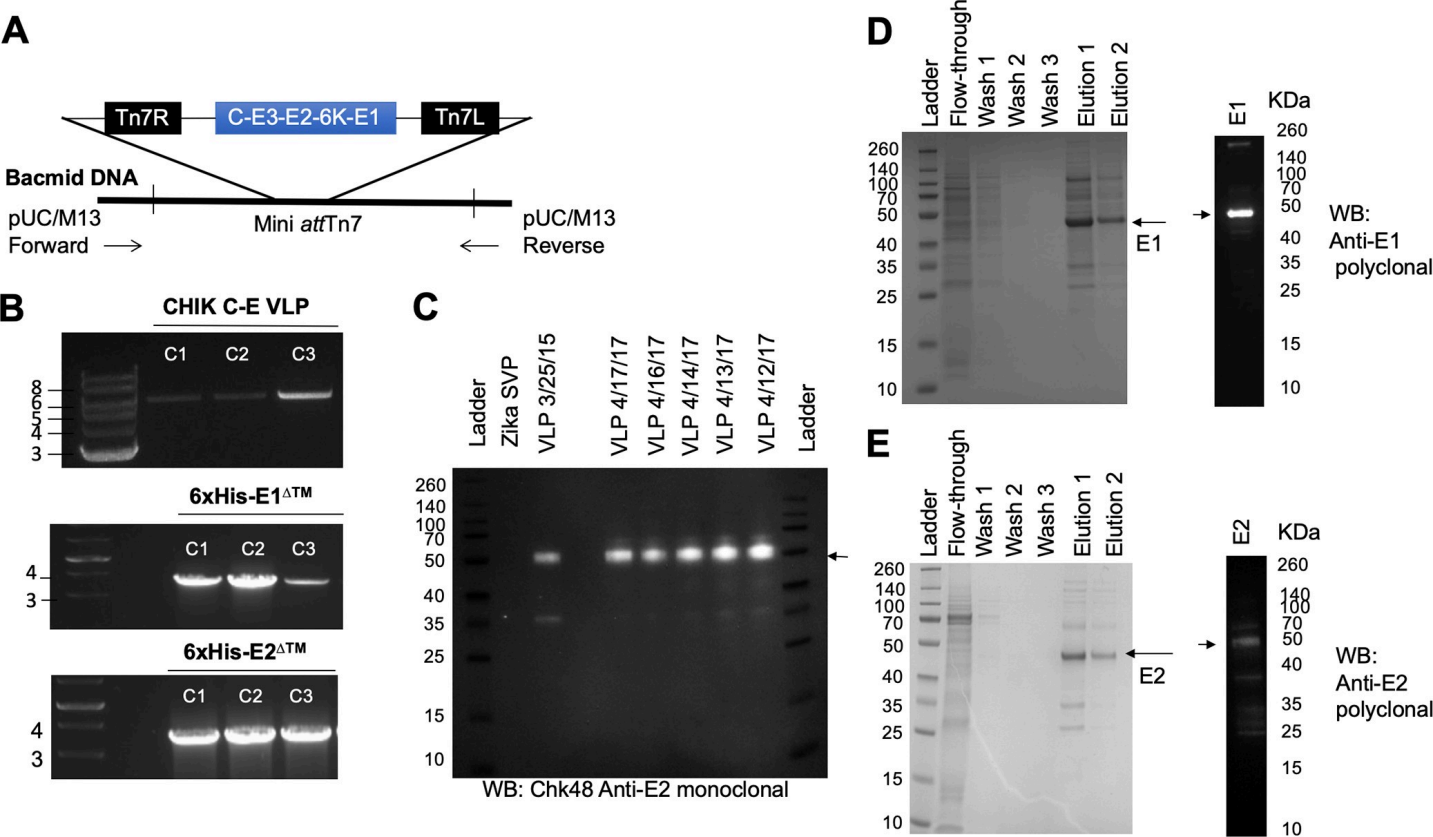
Chikungunya E and VLP design and verification. A] Schematic representation of CHIK gene insertion into bacmid. B] PCR verification of C-E; truncated, N-terminal his-tagged E1, and truncated, N-terminal his-tagged E2 clones. C] Expression of CHIK VLPs were verified by Western blot analysis using anti-E Chk48 monoclonal antibody. D] Soluble, his-tagged E1 was purified via chromatography using NiNTA agarose beads and the different fractions were analyzed via SDS-PAGE with PageBlue protein staining solution. Purified, soluble E1 was detected by western blot using anti-E1 polyclonal sera generated in C57BL/6J mice. E] Soluble, his-tagged E2 was purified via chromatography using NiNTA agarose beads and the different fractions were analyzed via SDS-PAGE with PageBlue protein staining solution. Purified, soluble E2 was detected by western blot using anti-E2 polyclonal sera generated in C57BL/6J mice.

CHIK VLPs, as well as CHIKV E1 and E2 proteins were produced and purified, as vaccines or reagents to analyze the immune responses elicited against CHIKV antigens. Multiple AcMNPV bacmids were generated to encode CHIKV C-E genes for expression and subsequent VLP assembly and for 6×His-E1^ΔTM^ and 6×His-E2^ΔTM^. CHIKV gene insertions were verified by PCR analysis [**Fig 1A**]. Three bacmid clones (c1-c3) were chosen for each construct. The C-E [3765 bp] insert plus flanking sequences (2300 bp) resulted in a 6065 bp band and all three CHIK C-E VLP bacmid clones contained the correct insert as determined by electrophoresis through 1% agarose in tris-acetate-EDTA (TAE) [**Fig 1B, top**]. The bacmid clones containing E1^ΔTM^ (1266 bp) plus flanking sequences, including upstream HIS-tag region (2430 bp) resulted in a PCR product of 3696 bp and these constructs were also verified by gel electrophoresis [**Fig 1B, middle**]. The bacmid clones containing E2^ΔTM^ (1122 bp) plus flanking sequences, including upstream HIS-tag region (2430 bp) resulted in a PCR product of 3552 bp [**Fig 1B, bottom**]. These bacmids were independently used to transfect SF9 cells to successfully produce recombinant baculoviruses Ac-C-E VLP, Ac-6×His-E1^ΔTM^, and Ac-6×His-E2^ΔTM^ capable of expressing CHIKV VLP and E proteins [**Fig 1C–1E**].

VLPs self-assembled from C-E proteins and were recovered from Ac-C-E VLP-infected SF9 cell culture medium. These particles were purified from non-particles by ultracentrifugation through a 20% glycerol cushion, as we previously reported [21]. The purified particles were probed using the Chk48 anti-E2 monoclonal antibody and a 50 kDa band representing the CHIKV E2 protein was detected by Western blot [**Fig 1C**]. Five of six batches of purified VLPs [4/12/17-4/17/17] were pooled and used for vaccination. These lots were compared to a batch produced two years earlier [3/25/15] that were stored at -80°C, demonstrating stability of VLP over time, when frozen. Zika subviral particles (SVP) were not detected using the Chk48 anti-E2 antibody. Like the VLP, HIS-tagged E1 and E2 proteins were secreted into the culture media of Sf9 cells infected with their respective baculoviruses: Ac-6×His-E1^ΔTM^ and Ac-6×His-E2^ΔTM^. These E1 and E2 proteins were purified via affinity chromatography using NiNTA resin. For each set of purifications, unbound protein flow-through, three separate washes, and two separate elutions were collected and analyzed by SDS-PAGE, with PageBlue protein staining. Untagged proteins were removed by the second and third washes as shown by SDS-Page analysis [**Fig 1D and 1E**]. Recombinant E1 was successfully recovered in elutions 1 and 2, as demonstrated by the presence of an intense band at approximately the expected size of 48 KDa [**Fig 1D**]. E1 protein was recovered in the elutions and then pooled, dialyzed, and concentrated. Anti-E1 mouse polyclonal sera reacted strongly with a~48 kDa band by Western blot analysis. Elutions 1 and 2 containing a 44 kDa recombinant E2 protein [**Fig 1E**] were pooled together, dialyzed, and concentrated. Anti-E2 polyclonal sera recognized the purified, recombinant E2 band.

### Vaccination and CHIKV-specific antibody responses

**Fig 2:**
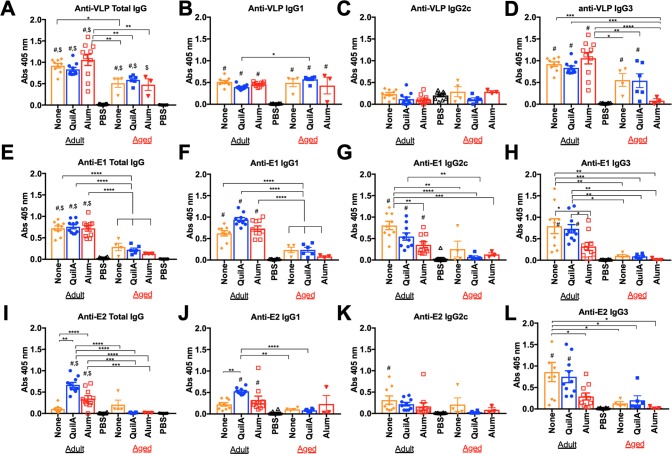
Chikungunya antigen-specific, IgG antibody responses in adult vs aged mice. Vaccine responses in adult and aged C57BL/6J mice were evaluated 8 weeks [three vaccine doses] post-initial vaccination with each VLP-based vaccine formulation delivered via intramuscular [IM] injections, or PBS. ELISAs were performed to evaluate anti-VLP responses: A] total IgG, B] IgG1, C] IgG2c, and D] IgG3. Anti-E1 responses are shown as E] total IgG, F] IgG1, G] IgG2c, and H] IgG3. Anti-E2 responses are shown as I] IgG, J] IgG1, K] IgG2c, and L] IgG3. Mean ± standard error [SEM] are reported. One-way, two-tailed ANOVA, followed by Tukey post-hoc tests were performed: # significantly different from adult PBS mice, $ significantly different from aged PBS mice, *p < 0.05, ** p < 0.01, *** p < 0.001, and **** p < 0.0001.

Adult C57BL/6J mice were vaccinated at weeks 0, 3, and 6 with VLP alone or coupled with one of three different adjuvants: QuilA, R848, and Imject Alum. Preliminary studies identified CHIK VLP, VLP plus QuilA, and VLP plus Alum formulations as the top vaccine candidates in adult mice when delivered intramuscularly based on CHIKV antigen-specific IgG responses [S1A-S1C Fig], neutralizing antibody responses [S1D Fig], and protection against CHIKV-associated arthritis [S1E Fig]. Thus, these top candidates were also assessed in aged mice, following the same regimen. All adult and aged miced vaccinated with CHIK VLP formulations seroconverted after 3 doses as determined by ELISA for anti-VLP total IgG [**Fig 2A**], and anti-VLP total IgG levels were statistically significant when compared to both, adult and aged PBS control sera. However, anti-VLP total IgG titers were significantly higher in adult mice vaccinated with VLP alone in comparison to aged mice vaccinated with VLP alone (abs 0.9211 to 0.512, p = 0.0439). Morever, adult vaccinated with VLP plus Alum had higher anti-VLP total IgG titers then any of the aged mice vaccinated with VLPs (all p < 0.01). The aged CHIK VLP-vaccinated mice were able to mount anti-VLP IgG1 responses that were comparable to adult CHIK VLP-vaccinated mice [**Fig 2B**], but significantly lagged in their anti-VLP IgG3 responses [**Fig 2D**]. Anti-VLP IgG2c responses were insignificant in CHIK VLP-vaccinated adult and aged mice [**Fig 2C**]. The IgG responses against CHIK E1 protein were robust in all adult mice vaccinated with CHIK VLPs, but were insignificant in aged mice vaccinated with CHIK VLPs [**Fig 2E–2H**]. For adult mice vaccinated with VLP alone, the anti-E1 IgG subclass responses was dominated by IgG2c [abs 0.805] and IgG3 (abs 0.792), followed by IgG1 (abs 0.621) antibodies. For adult mice vaccinated with VLP plus QuilA, the anti-E1 IgG subclass responses consisted of IgG1 (abs 0.943), followed by IgG3 (abs 0.732), and finally IgG2c (abs 0.547). In contrast, adult mice vaccinated with VLP plus Alum largely consisted of IgG1 antibodies [abs 0.733], less IgG2c [0.354], ad no IgG3. The strongest anti-E2 total IgG responses were elicited in adult mice vaccinated with VLP plus QuilA or VLP plus Alum [**Fig 2I**]. Vaccination with VLP plus QuilA in adult mice resulted in a strong anti-E2 IgG3 response [abs 0.751, **Fig 2L**], followed by anti-E2 IgG1 [abs 0.527, **Fig 2F**], but no significant anti-E2 IgG2c [**Fig 2G**]. In contrast, the VLP plus Alum vaccination only elicited significant anti-E2 IgG1 (abs 0.324) antibodies. Adult mice vaccinated with VLP alone elicited significant anti-E2 IgG3 (abs 0.861) and IgG2c antibodies (abs 0.313), but no significant anti-E2 IgG1. CHIK VLP vaccinations in aged mice did not elicit any significant anti-E2 IgG responses.

**Fig 3:**
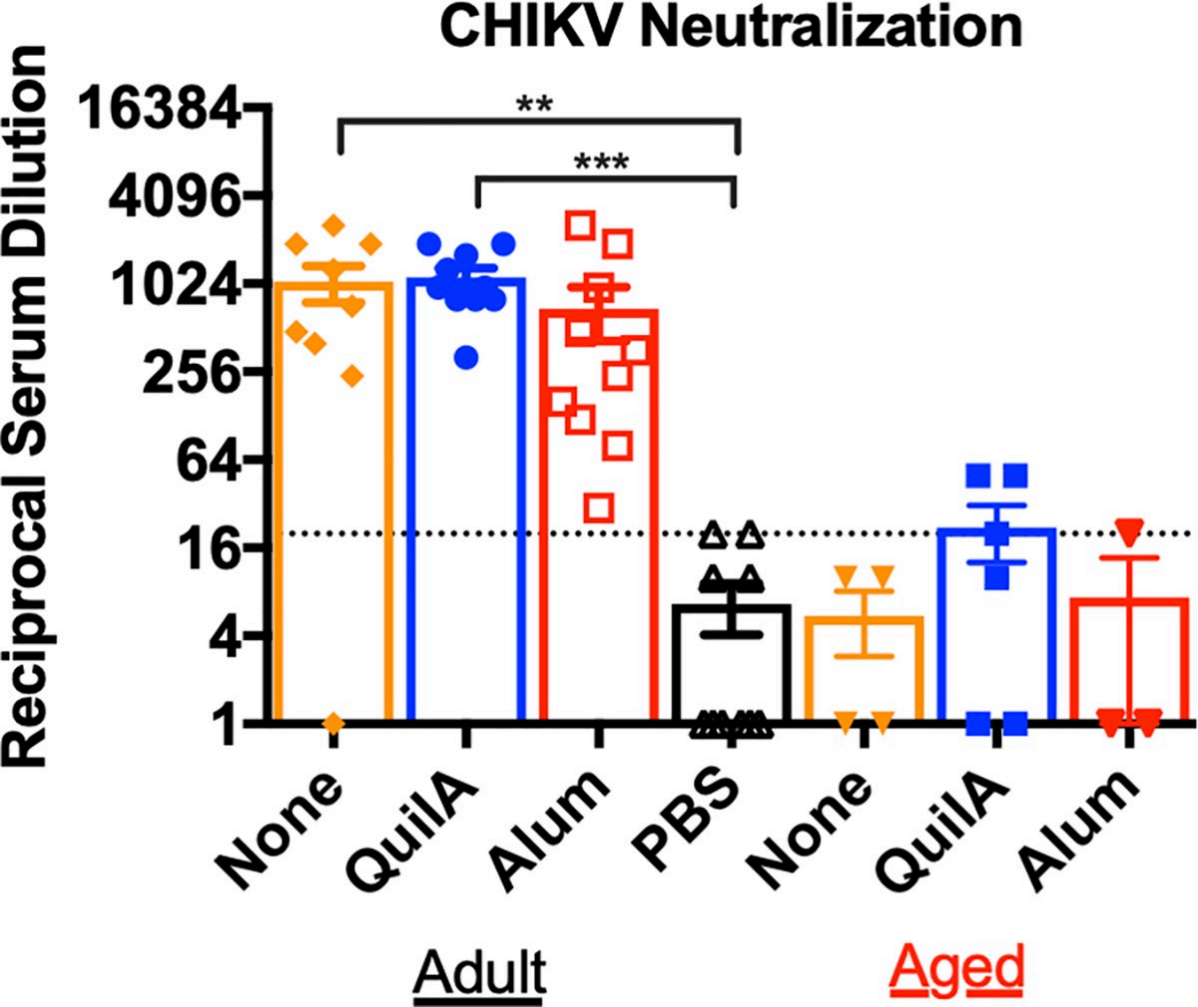
*In vitro* neutralization of CHIK LR2006-OPY1 by mouse immune sera. The ability of diluted sera from vaccinated mice to neutralize CHIKV LR2006-OPY1 infection of Vero cells was evaluated *in vitro*. The reciprocal serum dilution of the lowest dilution that prevented cytopathic effect in Vero cells is reported as the neutralization titer for individual mice, by group. Group neutralization titers are shown as mean ± S.E.M. The dotted line indicates the limit of detection of the assay. Kruskall-Wallis group analysis was performed for neutralization assay results, followed by Dunn’s multiple comparisons test [* p < 0.05, ** p < 0.01, *** p < 0.001, and **** p < 0.0001].

Vaccinations with CHIK VLP-based formulations induced antibodies in adult mice that were able to neutralize CHIKV LR2006-OPY1 [**Fig 3**, mean titer range 1:691–1:1136]. In adult mice, CHIK VLP only and CHIK VLP plus QuilA formulations elicted significant neutralization titers as compared to control sera. However, in aged mice, the VLP formulations failed to elicit CHIKV-neutralizing antibodies.

### Chikungunya viral challenge in adult mice vs aged mice

**Fig 4:**
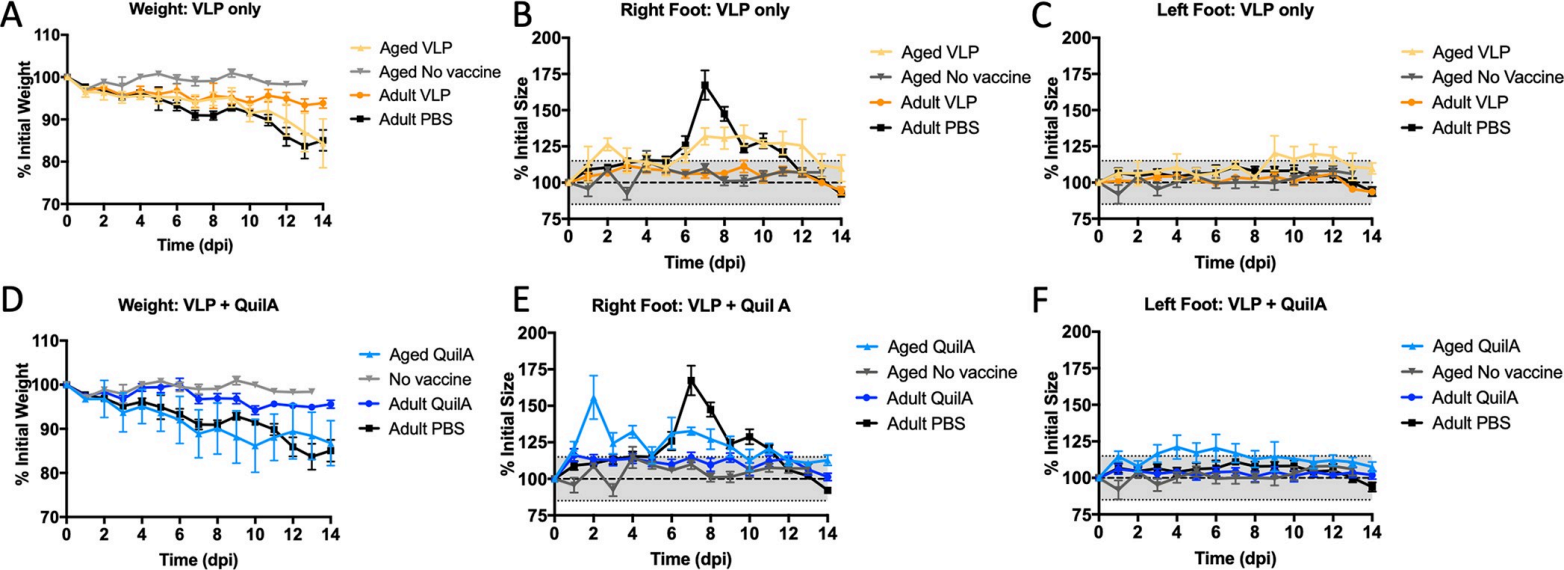
CHIKV-associated disease in adults vs aged mice. Adult and aged C57BL/6J mice were infected with 10^5^ CCID_50_ per mouse of CHIKV LR2006-OPY1 via subcutaneous injection of the right hind footpad and monitored for 14 days. A, D] Weights normalized to day 0 measurements are shown by vaccine formulation and age. B, E] Right foot size normalized to day 0 measurements are shown by vaccine formulation and age. C, F] Normalized left foot sizes are shown. The mean ± S.E.M are reported. The grey area loosely represents measurement variabilities observed in the non-infected left foot size of adult mice.

Mice were infected with 10^5^CCID_50_ CHIKV LR2006-OPY1 via subcutaneous injection of the right hind footpad. The infected mice were then monitored for 14 days following CHIKV infection. Blood samples were collected on days 2, 4, 6, and 14. While there was some gradual weight loss in the control adult mice, there was little weight loss observed in the adult mice vaccinated with CHIK VLPs only or CHIK VLPs plus QuilA [**Fig 4A and 4D**]. Unvaccinated aged mice did not lose any weight. However, aged mice vaccinated with CHIK VLPs or VLPs plus QuilA experienced some gradual weight loss, similar to what was observed in unvaccinated adult mice [**Fig 4A and 4D**].

We also measured the size of the infected right hindfeet and the uninfected left hindfeet were measured as additional controls. Any foot size deviation beyond 15% of the intitial foot size was considered a significant change. Adult mice vaccinated with PBS experienced significant swelling of the CHIKV-injected right hind foot. Swelling was visible by day 6 post-infection. Peak right foot swelling occurred on day 7, reaching approximately 170% of the initial foot size, before gradually returning to normal size by day 12 post-infection [**Fig 4B and 4E**]. Immunizations with VLP alone [**Fig 4B**] or VLP plus QuilA [**Fig 4E**] in adult mice offered complete protection throughout the complete time-course of the experiment. No measurable inflammation of the left hind feet were observed in any of the adult mice [**Fig 4C and 4F**]. In contrast to adult mice, naïve aged mice were resistant to CHIKV-mediated arthritis of the injected right hind foot [**Fig 4B and 4E**]. On the other hand, CHIK VLP-vaccinated old mice were more susceptible to CHIKV infection than naïve old mice. Aged mice vaccinated with VLP had pronounced foot swelling with one peak at day 2 post-infection [125% of initial foot size, **Fig 4B**], and then later on for a sustained period between 7–12 days post-infection (approximately 130% of initial foot size). There was significant right hind foot swelling in the aged group vaccinated with VLP plus QuilA on days 1–4 post-infection and again on days 6–9 [**Fig 4E**]. At peak swelling, the right foot size was 156% of its original size. There was little or no change in the size of left hind feet of challenged old mice change size over time [**Fig 4C and 4F**].

**Fig 5:**
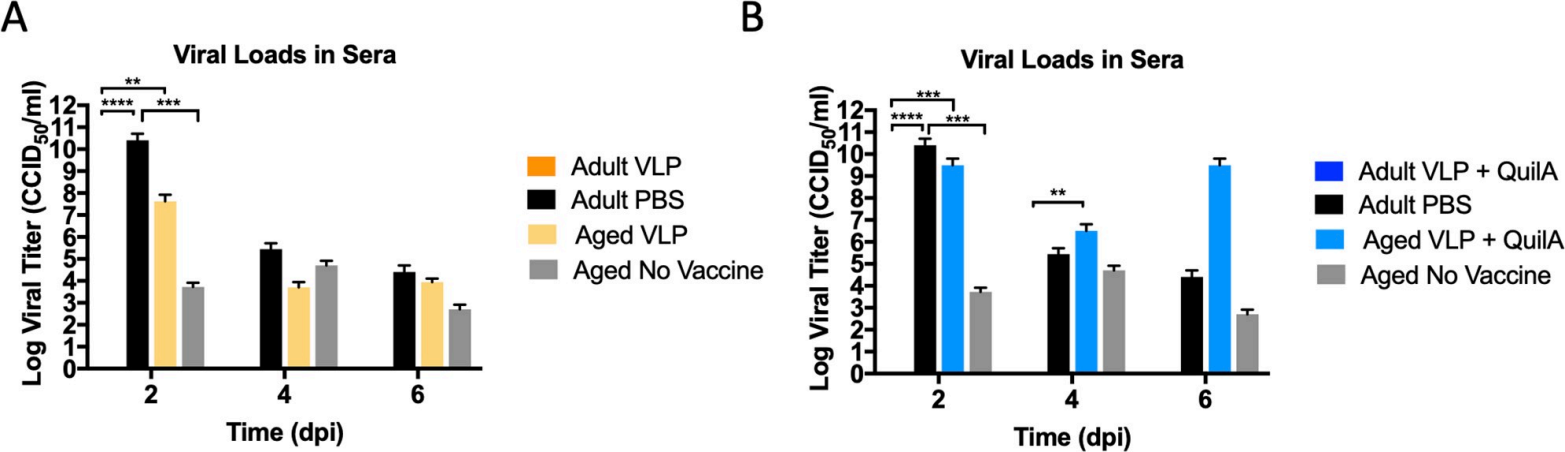
CHIK viral loads in adult vs aged mice. Infectious viral loads were determined from sera collected on days 2, 4, and 6 post-infection. A] Viral loads in mice vaccinated with VLP in comparison to control groups and B] Viral loads in mice vaccinated with VLP plus QuilA in comparison to control groups. The mean ± S.E.M are reported. One-way, two-tailed ANOVA, followed by Tukey post-hoc tests were performed: ** p < 0.01, *** p < 0.001, and **** p < 0.0001.

Infectious virus was recovered from adult mice vaccinated with PBS and challenged with CHIK LR2006-OPY1 at day 2 [2.53 × 10^10^ CCID_50_/ml], day 4 [2.77 × 10^5^ CCID_50_/.ml], and day 6 (2.52 × 10^4^ CCID_50_/.ml) **[Fig 5**]. Peak viral titers were observed on day 2 [**Fig 5**] and virus was completely cleared by day 14 post-infection. Virus was not recovered in adult mice vaccinated intramuscularly with VLP or VLP plus QuilA [**Fig 5A** and **5B**, respectively] at 2, 4, or 6 days post-infection. Viral infection in unvaccinated old mice produced significantly lower viral titers on day 2 [5.31×10^3^ CCID_50_/ml], with infection peaking on day 4 [5.08×10^4^ CCID_50_/ml], before decreasing on day 6 [507 CCID_50_/ml], and eventual clearance by day 14 [**Fig 5**]. In contrast, old mice vaccinated with VLP developed higher viremia [4.2×10^7^ CCID_50_/ml, **Fig 5A**] than unvaccinated old mice as measured on day 2 post-infection. However, viral loads in old, VLP-vaccinated mice decreased to similar levels as unvaccinated mice on days 4 and 6 post-infection. Old mice vaccinated with VLP plus QuilA also had higher viremia on day 2 post-infection (3.15×10^9^ CCID_50_/ml) than unvaccinated mice. The viral loads in old mice vaccinated with VLP plus QuilA decreased on day 4, but peaked again on day 6 post-infection [3.15×10^9^ CCID_50_/ml, **Fig 5B**].

**Fig 6:**
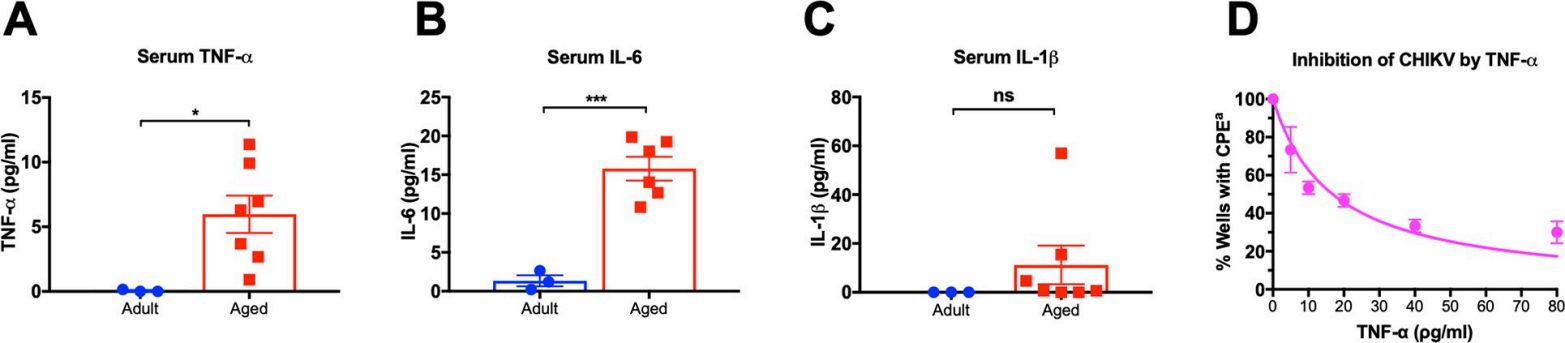
Baseline inflammation in the aging. Pools of naïve sera from adult or aged mice were tested for inflammatory cytokines: A] TNF-α, B] IL-6, and C] IL-1β. Two tailed t-tests were performed between adult and aged groups and p < 0.05 was considered significant (* p < 0.05, *** p < 0.001). D] Vero cells were infected with 200 CCID_50_ in the presence of TNF-α at range of 5–80 ρg/ml. ^a^ Wells were counted if there was at least 95% CPE.

The resistance to CHIKV infection observed in naïve, aged mice may be associated with chronic low-grade inflammation that accompanies aging [22]. To test this theory, sera, collected from naïve adult mice and aged healthy mice, were assayed for the presence of TNF-α, IL-6, and IL-1β [**Fig 6**]. TNF-α, IL-6, and IL-1β were below levels of detection in healthy, naïve, adult mice. Pooled sera from groups of aged mice that appeared otherwise healthy had significantly elevated basal levels of TNF-α at 5.971 ± 3.82 pg/ml in comparison with adult mice [**Fig 6A**]. Aged mice also had significantly elevated basal levels of IL-6 at 15.17 ± 3.766 pg/ml in comparison to adult mice [**Fig 6B**]. Serum cytokine levels of IL-1β were not significantly altered in aged mice versus adult mice [**Fig 6C**]. However, two groups of pooled sera from healthy, aged mice had high IL-1β levels of 15.4 and 56.9 pg/ml. Overall, naïve aged mice have elevated basal levels of inflammatory cytokines in comparison to naive adult mice. Moreover, the presence of TNF-α inhibited infection of Vero cells infected with CHIKV LR2006-OPY1 as measured by reduced CPE in the monolayers with as little as 5 pg/ml [**Fig 6D**], and an IC_50_ of 14.49 ± 2.99 as determined by nonlinear regression using GraphPad Prism software.

## Discussion

Two of the most promising vaccines against CHIKV infection, a CHIK VLP vaccine and a measles-vectored vaccine expressing CHIK VLPs, have cleared Phase I trials with positive outcomes. These vaccines 1] are overall safe and tolerable, and 2] elicit CHIKV neutralization titers in adults 18–50 years of age. These two candidates are now being tested in healthy adults of 18–60 years of age in five Caribbean island nations. Based on results from Phase I trials, at least two immunizations with the live-vectored or CHIK VLPs are needed for 100% seronconversion and induction of neutralizing antibodies in healthy adult subjects [23, 24]. The goals of our study were to improve these vaccines by adding adjuvants, and also show that these formulations would be efficacious in an aged model of infection. R848/resiquimod was used as a TLR7/8 agonist to induce a Th1 biased response [25]. Imject Alum induces primarily a Th2 biased response [26] and QuilA was selected because it enhances T-dependent and T-independent immune responses [27]. In adult mice, CHIK VLP administered alone or with QuilA administered by IM elicited strong antibody responses that were neutralizing *in vitro*, and these vaccines protected 100% of mice against CHIKV challenge, as determined by lack of swelling of the injected foot, lack of weight loss, and lack of viral titers.

Antibodies play a critical role in the clearance of CHIKV infections by both neutralizing virus infection and enhancing the clearance of virally infected cells [28, 29]. Potent neutralizing antibodies, composed of multiple subclasses, are directed against the E2 protein [30]. Early induction of anti-CHIKV anti-E2 human IgG3 subclass antibodies successfully clear CHIKV infections and lead to faster recovery. In adult mice, the most effective vaccine candidates were CHIK VLPs with no adjuvant or VLP plus QuilA, both administered by IM injections. Both of these vaccine formulations elicited antibodies directed against both the E1 and E2 proteins. A combination of robust IgG1, IgG2c, and IgG3 anti-E1 responses were detected [**Fig 2F–2H**]. In contrast, IgG3 antibodies were the predominant anti-E2 response elicited by these vaccines, with lower titers of pro-inflammatory IgG2c elicited by VLP alone or IgG1 by VLP vaccines formulated with QuilA [**Fig 2J–2L**]. Mouse IgG3, which is not a homolog of human IgG3, is induced independently of T-cell help and appears shortly after vaccination [31]. This anti-CHIKV IgG3 response may enhance clearance of CHIKV infected cells in adult mice, since CD4 knockout mice are still able to control and clear CHIK virus as effectively as wild-type mice [32]. In contrast, B cell deficient mice (μMT) remain persistently infected [33]. Mouse IgG3 binds FcγRI, but is thought to function primarily through activation of complement [34]. These anti-E1 and anti-E2 responses were absent in aged mice vaccinated with CHIK VLPs or VLP plus QuilA. The sera from aged mice vaccinated did react with whole VLP preparations by ELISA, with similar levels of anti-VLP IgG1 as the adult mice. Perhaps the presence of these antibodies that did not bind specifically to soluble E1 or E2, contributed to CHIKV disease enhancement in these animals. A phenomenon coined antibody-dependendent enhancement has been described for dengue and other viruses whereby non-neutralizing antibodies facilitate entry of antibody-bound virions via FcγR [35]. Antibody-dependent enhancement has also been observed *in vitro* with another alphavirus, Ross River Virus [36]. A more recent study shows that convalescent sera from CHIKV-infected subjects mediates enhanced binding, but not enhanced replication of CHIKV in primary human monocytes and B cells *in vitro* via FcγRs [37]. In contrast, increased chikungunya viral replication is observed in Raw 264.7 mouse macrophages in the presence of mouse anti-CHIKV IgG *in vitro*. Mice infected with CHIKV and then treated with subneutralizing levels of anti-CHIKV IgG also develop higher levels of viremia and disease as measured by foot swelling [37].

Another unexpected feature of the aged mice was their resistance to chikungunya viral infection and disease [**Fig 5**]. We speculate that this resistance may be associated with elevated levels of inflammatory cytokines present in aged mice, but not in adult mice. Chronic, low-level inflammation associated with aging has been coined “inflammaging”. The elevation of some cytokines, such as IL-6, are associated with longevity, while elevation of other pro-inflammatory cytokines, such as TNF-α, are associated with higher rates of mortality in humans [22]. The role of inflammaging is complicated because while enhanced inflammation leads to disease, mortality, and poor vaccine outcomes, inflammation can help the immune system resist virally-induced disease [22]. Healthy aged mice with detectable levels of inflammatory markers TNF-α and IL-6 [**Fig 6A and 6B**] were resistant to CHIKV-associated disease [**Fig 4B & 4E, Fig 5]**. Furthermore, addition of exogenous TNF-α to Vero cells inhibited CHIKVinfection *in vitro*, based on reduction in CPE. Future studies testing the effects of exogenous TNF-alpha on CHIK viral infections *in vivo* would help to corroborate these results.

Basal inflammation in the naïve, aged mice may have been helped these mice resist CHIKV infection, but it may also have contributed to the poor immune responses to vaccination with CHIK VLPs. Reduction of vaccine efficacy due to inflammation has been observed in elderly people vaccinated with standard vaccines against influenza virus [38] and hepatitis B [39]. Furthermore, elevated plasma levels of TNF-α correlate with lower antibody titers generated in post-menopausal women following vaccination with influenza vaccine. Thus, it is possible that the basal levels of TNF-α observed in age mice, but not in young mice, contributed to the decrease antibody responses observed after vaccination with CHIK VLP-based vaccines. A recent publication suggests that this problem may be circumvented by pre-treament of elderly patients with anti-inflammatory drugs, such as Losmapimod, a small molecule p38 mitogen-activated protein kinase inhibitor [40]. In addition, given that elderly people are already in a pro-inflammatory state, using an adjuvant to enhance inflammatory responses to a vaccine may not be the most beneficial approach for CHIKV.

In contrast to our observations in CHIKV-infected aged mice, a study by Uhrlaub *et al*. [41] found that CHIKV infections resulted in more severe infections in aged mice. There are a few key differences between our studies. The ages of the adult and aged mice were similar, but we used female mice, while Uhrlaub *et al*. used male mice. They also used a different CHIKV strain: SL15649. This strain resulted in a different disease progression in adult male mice than what we observed in adult female mice: foot-swelling was biphasic with two peaks on days 3 and then on day 8 with SL15649 infection, while we observed one main peak on day 7 with LR2006-OPY1. While the progression of CHIKV-associated foot swelling we observed is comparable to what has been previously published by Gardner et al [19] and Metz et al [42] using CHIKV LR2006 OPY1 strain, biphasic foot-swelling has also been in observed with this same strain at 10^6^ CCID_50_ in female mice [43] and at 10^3^ focus-forming units in female and male mice [44]. Thus, the use of different strains cannot be solely responsible for the difference in results. While not previously anticipated in our study, the stark difference in results may have to do with gender in aged mice. A longitudinal, retrospective study on prognostic factors of inhospital deaths in elderly patients by L. Godaert *et al*. [45] found that the male sex was an independent predictor of inhospital deaths due to CHIKV infection. Thus, perhaps in aged mice, the male sex may also predispose them to more severe disease. In addition study by Uhrlaub *et al*. also showed that CD4+ T cells and neutralizing antibody responses elicited by CHIKV infections were significantly decreased in aged, male mice compared to the adult, male mice. Thus overall, even in the male mice, the VLP vaccine formulations would likely also elicit poor protective responses as compared to those in adult mice.

While our study suggests that naïve, aged, female mice are resistant to CHIKV infections, infections in aged people are much more complicated. Comorbidities may increase the risk of developing more severe CHIKV disease upon infection. CHIKV infection may be complicated by pre-exisiting comorbidities or may exacerbate chronic renal, respiratory, cardiac diseases [9, 46]. A recent systematic meta-analysis of 11 different studies showed that hypertension, diabetes, cardiac disease, and asthma were the most frequent comorbidities associated with patients infected with CHIKV [47]. Furthermore, hypertension and diabetes had a 4-5-fold higher prevalence in patients over 50 years of age, and patients with diabetes at higher risk for severe CHIKV disease [47]. Thus, perhaps CHIKV infections in diabetic mouse or non-human primate models may provide a better understanding of CHIKV infection, spread, and virus-induced disease pathology observed in severe CHIKV-induced disease. In addition, it would be important to investigate these preconditions in both female and male animals. These models could then be used for preclinical testing of vaccines and antivirals against CHIKV.

In summary, CHIK VLPs alone elicit strong neutralizing antibody titers that protect 100% of mice from CHIKV infection and disease. However, more research is needed to identify a vaccine that will protect the elderly and people with chronic conditions, such as hypertension and diabetes against CHIKV disease. If the current measles-vectored an CHIK VLP-based vaccines make it through all phases of clinical trials, licensing of these vaccines should be limited to the aged groups in which they were tested in, until further research can be conducted. It is possible that a completely different set of vaccine formulations or regimens may need to be developed for the elderly.These would need to be thoroughly vetted in preclinical studies to ensure that older people are not put at further risk for severe CHIKV infections. Finally, relevant models of severe CHIKV disease in adults and aged animals are needed to evaluate these vaccines.

## Supporting information

S1 Fig Initial Assessment of Chikungunya antigen-specific, IgG antibody responses in adult mice. Vaccine responses in adult C57BL/6J mice were evaluated 8 weeks [three vaccine doses] post-initial vaccination with each VLP-based vaccine formulation delivered via IM injections, or PBS. ELISAs for total IgG were performed to evaluate A] anti-VLP, B] anti-E1, and C] anti-E2 responses. D] The ability of diluted sera from vaccinated mice to neutralize CHIKV LR2006-OPY1 infection of Vero cells was evaluated *in vitro*. The reciprocal serum dilution of the lowest dilution that prevented cytopathic effect in Vero cells is reported as the neutralization titer for individual mice, by group. Mean ± standard error (SEM) are reported. One-way, two-tailed ANOVA, followed by Tukey post-hoc tests were performed: # significantly different from adult PBS mice, *p < 0.05, ** p < 0.01, *** p < 0.001, and **** p < 0.0001. E] Mice were infected with 10^5^ CCID_50_ per mouse of CHIKV LR2006-OPY1 via subcutaneous injection of the right hind footpad and monitored for 14 days. Right foot size normalized to day 0 measurements are shown by vaccine formulation (Mean ± SEM shown).
(PDF)
