# Supplementary material for: Vaccination with a chikungunya virus-like particle vaccine exacerbates disease in aged mice
Source: PLoS Negl Trop Dis. 2019 Apr 26;13(4):e0007316. doi: 10.1371/journal.pntd.0007316 (PMC6485612; doi:10.1371/journal.pntd.0007316)
Supplement: S1 Fig — Vaccine responses in adult C57BL/6J mice were evaluated 8 weeks [three vaccine doses] post-initial vaccination with each VLP-based vaccine formulation delivered via IM injections, or PBS. ELISAs for total IgG were performed to evaluate A] anti-VLP, B] anti-E1, and C] anti-E2 responses. D] The ability of diluted sera from vaccinated mice to neutralize CHIKV LR2006-OPY1 infection of Vero cells was evaluated in vitro. The reciprocal serum dilution of the lowest dilution that prevented cytopathic effect in Vero cells is reported as the neutralization titer for individual mice, by group. Mean ± standard error (SEM) are reported. One-way, two-tailed ANOVA, followed by Tukey post-hoc tests were performed: # significantly different from adult PBS mice, *p < 0.05, ** p < 0.01, *** p < 0.001, and **** p < 0.0001. E] Mice were infected with 105 CCID50 per mouse of CHIKV LR2006-OPY1 via subcutaneous injection of the right hind footpad and monitored for 14 days. Right foot size normalized to day 0 measurements are shown by vaccine formulation (Mean ± SEM shown). (PDF) [file pntd.0007316.s001.pdf]

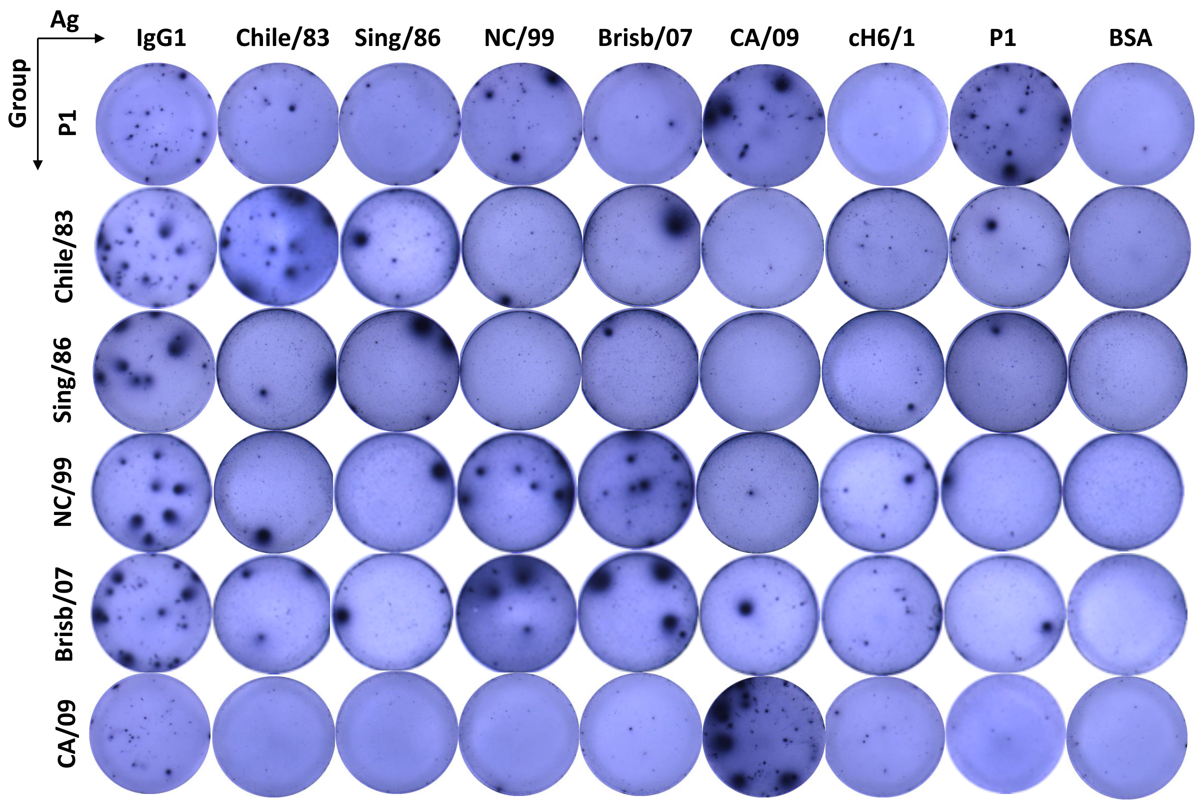

Supplementary Figure 1. Representative ELISPOT wells and percentage of IgG1 or Ag-specific IgG1 secreting cells. ~$1.7x10^5$ viable splenocytes from P1-, Chile/83-, Sing/86-, NC/99-, Brisb/07- and CA/09-immunized mice were plated into representative wells with the exception of those for IgG1 and homologous rHA, in which ~$1.9x10^4$ cells were plated. Data were generated in a single experiment and are representative of three independent experiments evaluating the breadth of IgG1 secreting cells. For each independent experiment, splenocytes from the same mouse were run in triplicate.

rHA	% of IgG1 SC (% of HA+ IgG1 SC)					
	**P1 group**	**Chile/83 group**	**Sing/86 group**	**NC/99 group**	**Brisb/07 group**	**CA/09 group**
**P1**	6.29% (100%)	0.44% (9.49%)	0.23% (3.81%)	0.63% (10%)	0.33% (4.85%)	0.37% (5.82%)
**Chile/83**	0.89% (14.11%)	4.65% (100%)	0.17% (2.86%)	0.54% (8.61%)	1.49% (21.82%)	0.18% (2.79%)
**Sing/86**	0.71% (11.3%)	0.4% (8.72)	5.99% (100%)	0.61% (9.72%)	0.7% (10.3%)	0.36% (5.59%)
**NC/99**	2.12% (33.73)	0.15% (3.13%)	0.05% (0.86%)	6.32% (100%)	1.87% (27.27%)	0.12% (1.86)
**Brisb/07**	1.1% (17.52%)	0.32% (6.92%)	0.34% (5.71%)	1.79% (28.33%)	6.85% (100%)	0.36% (5.59%)
**CA/09**	2.94% (46.72%)	0.42% (8.97%)	0.06% (0.95%)	0.42% (6.67%)	0.46% (6.67%)	6.51% (100%)
**cH6/1**	0.79% (12.48%)	0.25% (5.38%)	0.06% (0.95%)	0.44% (6.94%)	0.44% (6.42%)	0.63% (9.77%)

Supplementary Table I. Percentages of IgG1 secreting cells (SC) recognizing the different tested rHA through ELISPOT assay. In parenthesis are shown the percentages of IgG1 SC relative to the rHA-specific cells.

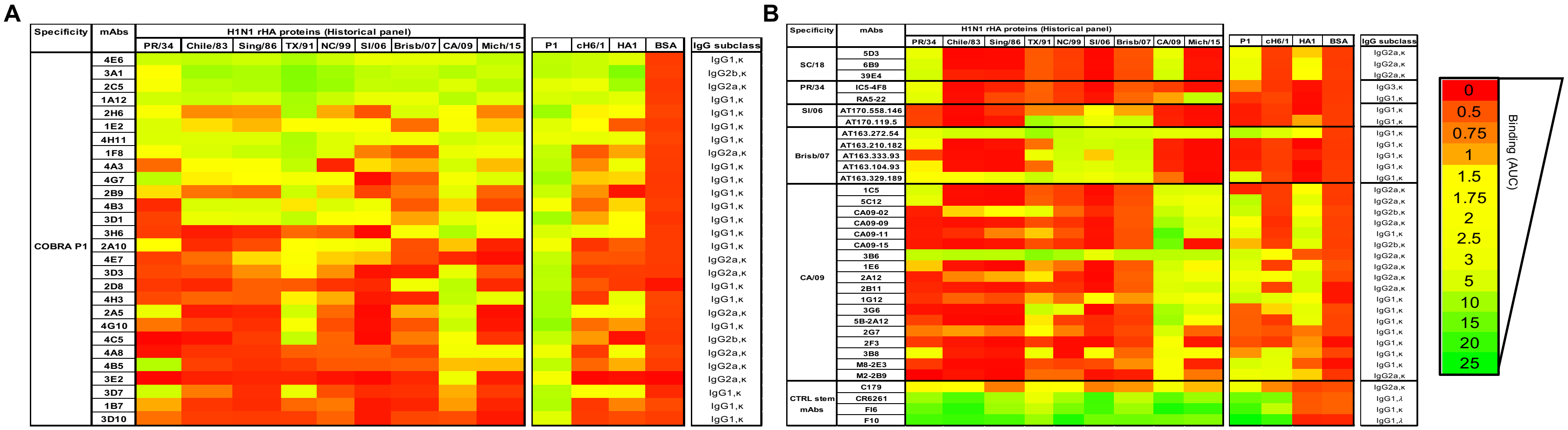

Supplementary Figure 3. Heatmap of COBRA P1, seasonal, pandemic and control stem-directed mAbs breadth of binding against recombinant H1N1 HA. Binding of COBRA P1 (**A**), seasonal (PR/34, SI/06, Brisb/07), pandemic (SC/18 and CA/09) and control stem-directed (**B**) mAbs was evaluated against a panel of H1 recombinant HA (rHA) from historical seasonal (PR/34, Chile/83, Sing/86, TX/91, NC/99, SI/06, Brisb/07), pandemic (CA/09) and post-pandemic (Phil/13 and Mich/15) rHA. Binding activity was also tested against COBRA P1 rHA, the chimeric (cH6/1) rHA and a truncated HA1 rHA. For COBRA P1, SC/18, CA/09 and control stem mAbs the CA/09 HA1 was used while for PR/34 and SI/06 or Brisb/07 mAbs a PR/34 and a Brisb/07 HA1 was used, respectively. All the mAbs were tested at different 3-fold dilutions, starting from 20 μg/mL. The area under the curve (AUC) was calculated from each dilution curve and reported on the heatmap table.
